# Emotional labor and occupational well-being among endoscopy healthcare workers: a moderated serial mediation model

**DOI:** 10.3389/fpubh.2026.1847595

**Published:** 2026-06-15

**Authors:** Zhi Zeng, Yazhi He, Xiang Liao, Sumei Zhou

**Affiliations:** 1 Endoscopy Center, Deyang People’s Hospital, Deyang, Sichuan, China; 2 Department of Neurosurgery, Deyang People’s Hospital, Deyang, Sichuan, China

**Keywords:** emotional labor, endoscopy healthcare workers, job control, moderated serial mediation, occupational well-being, organizational support, work engagement

## Abstract

**Background:**

Occupational well-being among healthcare professionals is a key indicator of healthcare quality and workforce sustainability. However, the mechanisms linking emotional labor to occupational well-being among endoscopy healthcare workers remain insufficiently understood.

**Objective:**

This study aimed to examine the relationship between emotional labor and occupational well-being and to explore the underlying mechanisms using a moderated serial mediation model.

**Methods:**

A multicenter cross-sectional survey was conducted between January and February 2026 among 364 endoscopy healthcare workers in China. Validated instruments were used to assess emotional labor, job control, work engagement, organizational support, and occupational well-being. Pearson correlation, regression analysis, and bootstrap methods were employed to test mediation and moderation effects.

**Results:**

Emotional labor was significantly negatively associated with occupational well-being (*r* = −0.325, *p* < 0.001). Job control and work engagement played significant mediating roles in this relationship. Specifically, the indirect effect through job control was significant (effect = −0.119, 95% CI: −0.182 to −0.056), as was the indirect effect through work engagement (effect = −0.046, 95% CI: −0.091 to −0.001). In addition, a significant serial mediation effect was observed through job control and work engagement (effect = −0.107, 95% CI: −0.163 to −0.051), with a significant total indirect effect (effect = −0.272, 95% CI: −0.380 to −0.164). Organizational support significantly moderated the relationship between emotional labor and job control (β = 0.018, *p* < 0.001), such that higher levels of organizational support attenuated the negative effect of emotional labor on job control.

**Conclusion:**

Emotional labor is associated with occupational well-being among endoscopy healthcare workers through correlational pathways. Job control and work engagement serve as key mediating mechanisms, while organizational support functions as an important buffering resource. Interventions aimed at reducing excessive emotional labor demands and strengthening job resources may contribute to improved occupational well-being among healthcare professionals.

## 1 Introduction

In the healthcare system, occupational well-being among medical staff is widely regarded as a key indicator for assessing the operational quality of healthcare organizations and the health status of human resources (1). Occupational well-being not only reflects healthcare professionals’ subjective evaluation of their professional value and work conditions, but is also closely associated with psychological health, job satisfaction, work performance, burnout, and the quality of patient care (2, 3).

In recent years, with the increasing demand for healthcare services and the rapid advancement of medical technologies, healthcare professionals have been subjected to continuously rising work pressures. As a result, issues related to occupational well-being have gradually become a critical concern in the fields of healthcare management and public health (4). Previous studies have demonstrated that higher levels of occupational well-being can significantly enhance work engagement and organizational commitment among healthcare professionals, thereby improving the quality of healthcare services and promoting the sustainable development of healthcare systems (5, 6). Therefore, exploring the key factors influencing occupational well-being among medical staff is of significant importance.

Emotional labor constitutes an essential component of healthcare professionals’ daily work. Hochschild defined it as a form of labor in which individuals regulate and manage their internal emotional experiences and external emotional expressions in the work process to comply with organizational display rules and role requirements (7).

In interactions with patients and their families, healthcare professionals are required to consistently express care, patience, and empathy, even when facing heavy workloads or complex doctor–patient relationships, while maintaining a stable and positive emotional state (8). Previous studies have shown that emotional labor exerts a dual impact on individuals’ occupational experiences: moderate emotional regulation can enhance job satisfaction and professional identity, whereas prolonged exposure to high levels of emotional labor may lead to emotional exhaustion and burnout (9).

In the context of endoscopy centers, where work is characterized by a fast pace, complex technical procedures, and frequent emotional interactions, medical staff are often required to engage in high levels of emotional labor (10, 11). Therefore, emotional labor may have a significant impact on their occupational well-being. Accordingly, the following hypothesis is proposed: H1: Emotional labor has a significant effect on occupational well-being among medical staff in endoscopy centers.

Job control refers to an individual’s subjective perception of autonomy over task arrangements, work pace, and work methods during the work process, and is considered an important psychological resource (12). According to the Job Demands–Resources (JD-R) model (13) and Conservation of Resources (COR) theory (14), excessive job demands and sustained emotional regulation may progressively consume individuals’ psychological resources. COR theory further emphasizes that individuals strive to obtain, maintain, and protect valuable resources, whereas continuous emotional demands may lead to resource depletion and resource loss, thereby weakening their sense of control over the work environment. When healthcare professionals are exposed to prolonged high levels of emotional labor, they are required to continuously regulate and manage their emotions. This process of emotional regulation is often accompanied by the consumption of psychological resources, which in turn reduces their perceived control over work processes (15). Previous studies have shown that high levels of emotional labor are significantly negatively associated with Job control, while higher levels of job control help individuals maintain positive psychological states and enhance job satisfaction (16, 17). Therefore, the following hypothesis is proposed: H2: Job control mediates the relationship between emotional labor and occupational well-being.

Work engagement refers to a positive psychological state characterized by vigor, dedication, and absorption during the work process, and is considered an important psychological mechanism influencing work outcomes (18). Previous studies have indicated that emotional labor requires individuals to continuously regulate and manage their emotions, a process that consumes psychological resources and may consequently reduce their level of work engagement (19). At the same time, higher levels of work engagement contribute to improved job satisfaction and occupational well-being (20). Research has also shown that Job control can enhance work engagement, and that higher levels of work engagement further promote the development of occupational well-being (21). In addition, work engagement has been found to play a significant mediating role in the relationship between job stress or job demands and occupational well-being (20, 22). Therefore, the following hypotheses are proposed: H3: Work engagement mediates the relationship between emotional labor and occupational well-being. H4: Job control and work engagement jointly play a serial mediating role in the relationship between emotional labor and occupational well-being.

Perceived organizational support refers to employees’ overall perception of the extent to which the organization values their contributions and cares about their well-being, and it is considered an important organizational resource (23). According to social exchange theory, when individuals perceive a high level of organizational support, they are more likely to develop positive work attitudes and stronger emotional attachment to the organization (24). Previous studies have shown that organizational support not only directly enhances employees’ job satisfaction and psychological well-being, but also buffers, to some extent, the negative impact of work stress on individuals’ psychological resources (25, 26). Within the integrated framework of the JD-R model and COR theory, organizational support, as an important organizational resource, is more likely to exert its buffering effect during the early stage of the resource depletion process. Therefore, in the present study, organizational support was hypothesized to moderate the relationship between emotional labor and job control, rather than the latter stages of the mediation pathway. When employees face high job demands or emotional labor pressures, organizational support can strengthen their sense of security and resource perception at work (16). In contrast, when the level of organizational support is low, employees are more likely to experience resource depletion under high emotional labor demands, thereby reducing their sense of job control (27). Therefore, the following hypothesis is proposed: H5: In the proposed chain mediation model, organizational support moderates the relationship between emotional labor and job control.

**Figure 1:**
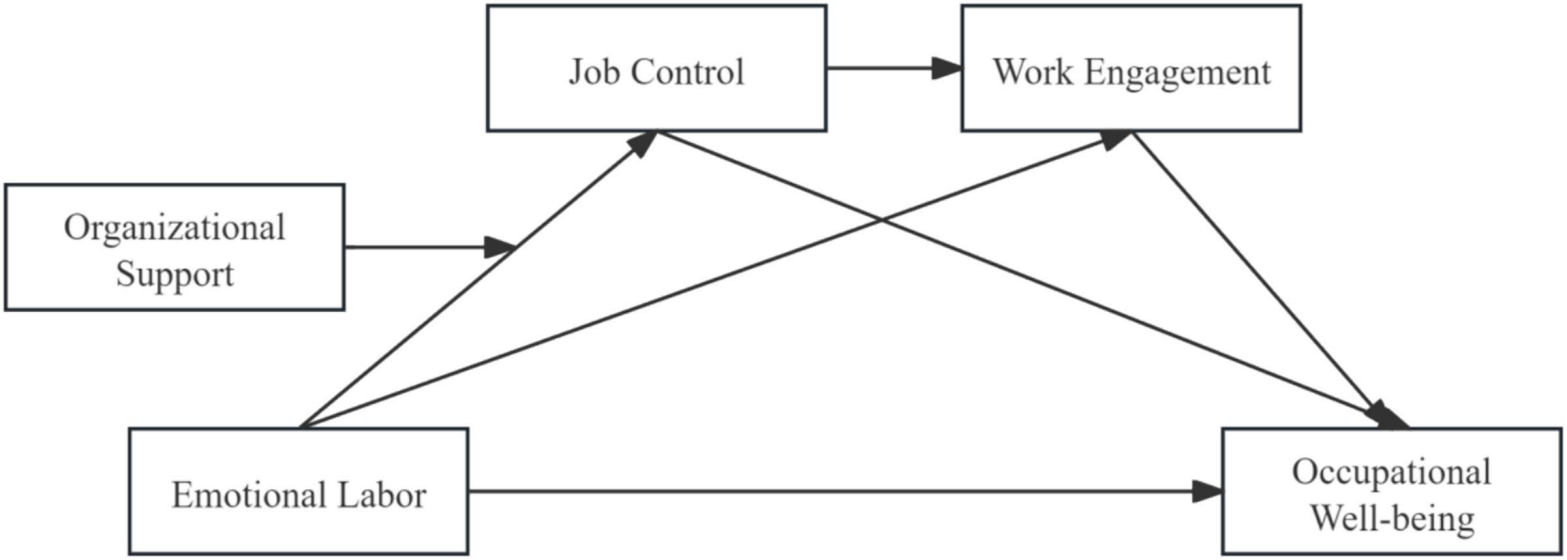
Hypothesized model.

Based on the above background, this study focuses on medical staff in endoscopy centers to examine the relationship between emotional labor and occupational well-being. Furthermore, a moderated serial mediation model was constructed to explore the serial mediating roles of job control and work engagement, as well as the moderating role of organizational support, in the relationship between emotional labor and occupational well-being. Furthermore, a moderated chain mediation model is constructed and tested to explore the underlying mechanisms through which emotional labor influences occupational well-being (Figure 1). The hypothesized model is presented as follows.

## 2 Methods

### 2.1 Study design and participants

This study employed a multicenter cross-sectional design and was conducted between January and February 2026 in endoscopy centers across multiple regions in China, including Sichuan, Zhejiang, Henan, Anhui, Shandong, Hubei, Jiangsu, Guangdong, and Chongqing. A convenience sampling method was used to recruit medical staff working in endoscopy centers, including physicians, nurses, and technical personnel.

The inclusion criteria were as follows: (1) having worked in an endoscopy center for at least 6 months; (2) possessing the required professional qualifications; and (3) providing informed consent and voluntarily participating in the study. The exclusion criteria included: (1) being on long-term leave (e.g., sick leave or maternity leave) during the survey period; (2) having experienced major life events or significant psychological stress recently; and (3) incomplete questionnaires or responses with obvious patterns.

This study was approved by the Ethics Committee of Deyang People’s Hospital (Approval No. 2023-04-083-K01).

### 2.2 Data collection

Data were collected using an online survey platform (Wenjuanxing). With the assistance of coordinators from participating hospitals, trained researchers distributed the questionnaire link and provided standardized instructions. All participants completed the survey anonymously and voluntarily after providing informed consent.

To ensure data quality, several measures were implemented, including mandatory responses, logical validation checks, and minimum completion time thresholds. Duplicate submissions and invalid responses were identified based on IP address screening and response patterns. All questionnaires were further reviewed manually, and those with logical inconsistencies were excluded. A total of 372 questionnaires were collected, of which 364 were valid, resulting in an effective response rate of 97.85%.

### 2.3 Measures

#### 2.3.1 Demographic and occupational characteristics

A self-developed questionnaire was used to collect participants’ demographic and work-related information, including gender, age, marital status, highest educational level, number of children, professional title, years of work in the endoscopy center, job category, average daily working hours, type of employing institution, and work shift arrangement.

#### 2.3.2 Occupational well-being scale for medical staff

Occupational well-being was assessed using the Occupational Well-being Scale for Medical Staff developed by Hu Dongmei et al. (28). The scale consists of 24 items covering five dimensions: physical and mental health, value/competence realization, social support, economic income, and work environment. A 5-point Likert scale was used, with responses ranging from 1 (“strongly disagree”) to 5 (“strongly agree”). The total score ranges from 24 to 120, with higher scores indicating higher levels of occupational well-being. The Cronbach’s α coefficient of the scale in this study was 0.93.

#### 2.3.3 Organizational support scale

Organizational support was measured using the scale developed by Eisenberger et al. (29). The scale consists of 8 items and has been widely applied across different occupational groups in China, demonstrating good structural validity and internal consistency. A 5-point Likert scale was used, with higher scores indicating higher levels of perceived organizational support. The Cronbach’s α coefficient of the scale in this study was 0.935.

#### 2.3.4 Job control scale

Job control was measured using the scale developed by Fox et al. (30) and translated and revised by Jia (31). The scale consists of 17 items and is unidimensional, assessing individuals’ perceived control over their work environment and work processes from multiple aspects. A 5-point Likert scale was used, with responses ranging from 1 (“very little”) to 5 (“very much”). Higher total scores indicate higher levels of job control. The Cronbach’s α coefficient of the scale in this study was 0.889.

#### 2.3.5 Work engagement scale

Work engagement was assessed using the short version of the Utrecht Work Engagement Scale (UWES-9) developed by Shimazu et al. (32). The scale consists of 9 items and includes three dimensions: vigor, dedication, and absorption. A 7-point Likert scale was used, with a total score ranging from 0 to 54. Higher scores indicate higher levels of work engagement. The Cronbach’s α coefficient of the scale in this study was 0.869.

#### 2.3.6 Emotional labor scale

Emotional labor was assessed using the Chinese version of the Emotional Labor Scale (ELS) revised by Luo et al. (33). The scale consists of 14 items and includes three dimensions: surface acting, deep acting, and natural expression. A 5-point Likert scale was used, with responses ranging from 1 (“strongly disagree”) to 5 (“strongly agree”). Higher scores indicate a higher frequency of using corresponding emotional labor strategies. In the present study, the total emotional labor score was used to reflect the overall level of emotional regulation demands experienced by healthcare workers. The Cronbach’s α coefficient of the scale in this study was 0.900.

### 2.4 Statistical analysis

Data were analyzed using Stata version 16.0. Continuous variables were expressed as mean ± standard deviation, and categorical variables as frequencies and percentages. Group differences were examined using independent-samples *t*-tests or one-way analysis of variance (ANOVA), while categorical variables were compared using chi-square tests. Pearson correlation analysis was conducted to examine the relationships among the main variables. Multicollinearity was assessed using variance inflation factors (VIFs), with VIF values below 10 indicating no serious multicollinearity.

Common method bias was assessed using Harman’s single-factor test. Confirmatory factor analysis (CFA) was conducted to evaluate construct validity, including model fit, convergent validity, and discriminant validity. Mediation and moderation effects were tested using bootstrap analyses with 5,000 resamples to estimate bias-corrected 95% confidence intervals. Educational level, professional title, years of work in the endoscopy center, job category, average daily working hours, and work shift arrangement were included as covariates in the adjusted analyses.

To further examine the proposed model, a moderated serial mediation analysis was conducted, with emotional labor as the independent variable, occupational well-being as the dependent variable, job control and work engagement as mediators, and organizational support as a moderator. Simple slope analysis was performed to interpret significant interaction effects. All tests were two-tailed, and *p* < 0.05 was considered statistically significant.

## 3 Results

### 3.1 Common method bias test

Common method bias was preliminarily assessed using Harman’s single-factor test. The results showed that the first factor accounted for 37.27% of the total variance, which is below the commonly used threshold of 40%. However, Harman’s single-factor test is only a preliminary diagnostic approach and cannot completely rule out the possibility of common method variance.

### 3.2 Reliability and validity

All study variables demonstrated satisfactory internal consistency, with Cronbach’s α coefficients exceeding 0.85. The Kaiser–Meyer–Olkin (KMO) values for all scales were above 0.75, and Bartlett’s test of sphericity was significant (*p* < 0.001), indicating suitability for factor analysis. In addition, all scales used in this study were previously validated Chinese versions that have demonstrated satisfactory psychometric properties in healthcare populations. Detailed results are presented in Table 1.

**Table 1:** Reliability and validity of the study variables.

Variables	Cronbach’s α	KMO	Bartlett’s test of sphericity		
			Approximate χ^2^	*df*	*P*
Occupational well-being	0.891	0.809	7862.431	276	0.001
Emotional labor	0.883	0.850	5583.471	91	0.001
Job control	0.940	0.806	5585.382	136	0.001
Work engagement	0.946	0.911	3222.812	36	0.001
Organizational support	0.943	0.903	3656.047	28	0.001

To further examine construct validity, confirmatory factor analysis (CFA) was conducted for the five-factor measurement model, including emotional labor, occupational well-being, job control, work engagement, and organizational support. The results indicated that the model demonstrated acceptable fit to the data (χ^2^/df = 2.84, CFI = 0.941, TLI = 0.932, RMSEA = 0.071, SRMR = 0.053). In addition, all standardized factor loadings were above 0.50 and statistically significant. The composite reliability (CR) values ranged from 0.89 to 0.95, and the average variance extracted (AVE) values ranged from 0.56 to 0.71, indicating satisfactory convergent validity. Furthermore, the square roots of the AVE values for all constructs exceeded the corresponding inter-construct correlations, supporting adequate discriminant validity.

### 3.3 Descriptive statistics and group differences

The results of the univariate analysis showed that occupational well-being differed significantly across categories of highest educational level, professional title, years of work in the endoscopy center, job category, average daily working hours, and work shift arrangement (all *p* < 0.001).

Specifically, individuals with associate senior titles or above, anesthesiologists, those working fewer than 8 h per day, and those working only day shifts reported relatively higher levels of occupational well-being. In contrast, those with junior titles, those working rotating shifts, primarily night shifts, or irregular schedules reported relatively lower levels of occupational well-being. Detailed results are presented in Table 2.

**Table 2:** Differences in occupational well-being across demographic characteristics.

Variables	Category	*N* (%)	Occupational well-being		*t/F*	*P*
			Mean	SD		
Gender	Male	70 (19.23)	84.543	11.95	1.410	0.236
	Female	294 (80.77)	82.51	13.099		
Age	≤30 years	25 (6.87)	83.44	10.215	0.300	0.828
	31–40 years	223 (61.26)	82.484	12.381		
	41–50 years	101 (27.75)	83.337	14.388		
	≥51 years	15 (4.12)	85.267	14.582		
Marital status	Unmarried	51 (14.01)	83.098	13.08	0.510	0.603
	Married	308 (84.62)	82.776	12.908		
	Divorced	5 (1.37)	88.6	11.014		
	Widowed	0 (0)	0	0		
Education level	Secondary school or below	6 (1.65)	86.833	10.778	19.720	0.001
	Junior college	29 (7.97)	99.172	17.823		
	Bachelor’s degree	303 (83.24)	81.419	11.562		
	Master’s degree or above	26 (7.14)	81.115	8.923		
Number of children	0	57 (15.66)	83.368	12.688	0.180	0.833
	1	188 (51.65)	82.505	11.834		
	≥2	119 (32.69)	83.303	14.583		
Professional title	Junior title	79 (21.7)	77.772	14.136	13.780	0.001
	Intermediate title	200 (54.95)	82.765	9.68		
	Associate senior title or above	85 (23.35)	87.988	16.126		
Years of work in the endoscopy center	<1 year	37 (10.16)	88.216	15.346	6.130	0.001
	1–3 years	47 (12.91)	82.915	9.401		
	4–6 years	85 (23.35)	77.6	13.394		
	7–10 years	71 (19.51)	83.268	11.478		
	>10 years	124 (34.07)	84.734	12.643		
Job category	Nurse	246 (67.58)	81.78	11.777	10.650	0.001
	Physician	72 (19.78)	81.625	10.72		
	Anesthesiologist	46 (12.64)	90.891	18.171		
Average daily working hours	<8 h	56 (15.38)	91.536	16.886	16.080	0.001
	8–10 h	292 (80.22)	81.308	11.555		
	>10 h	16 (4.4)	81.75	7.672		
Type of employing Institution	Public tertiary hospital	289 (79.4)	83.187	13.164	0.370	0.775
	Public secondary hospital	20 (5.49)	80.3	10.322		
	Private hospital	37 (10.16)	82.054	13.87		
	Community healthcare institution	18 (4.95)	82.944	8.788		
Work shift arrangement	Day shift	201 (55.22)	86.065	12.992	9.470	0.001
	Occasional night shifts	65 (17.86)	82.077	10.235		
	Rotating shifts	67 (18.41)	78.03	11.767		
	Irregular shifts	17 (4.67)	74.588	13.574		
	Irregular shifts	14 (3.85)	74.714	12.566		

### 3.4 Correlation analysis

Pearson correlation analysis showed that emotional labor was significantly negatively correlated with occupational well-being (*r* = −0.325, *p* < 0.001). In addition, occupational well-being was positively correlated with job control, work engagement, and organizational support (all *p* < 0.01).

Furthermore, emotional labor was negatively correlated with job control and work engagement, while job control was positively correlated with work engagement. These results provide preliminary support for the proposed mediation model (Table 3).

**Table 3:** Descriptive statistics and correlations among variables.

Variables	*M* ± SD	Occupational well-being	Emotional labor	Job control	Work engagement	Organizational support
Occupational well-being	82.901 ± 12.895	1				
Emotional labor	49.214 ± 7.687	−0.325***	1			
Job control	60.264 ± 10.601	0.320***	−0.240***	1		
Work engagement	36.047 ± 9.19	0.255***	−0.223***	0.318***	1	
Organizational support	37.984 ± 6.929	0.194***	0.262***	0.303***	0.135**	1

*p < 0.05, **p < 0.01, ***p < 0.001.

### 3.5 Serial mediation analysis

Prior to hypothesis testing, multicollinearity diagnostics were conducted. The results showed that all variance inflation factor (VIF) values were below 1.8, indicating that multicollinearity was not a concern and that the data were suitable for subsequent regression analysis.

**Figure 2:**
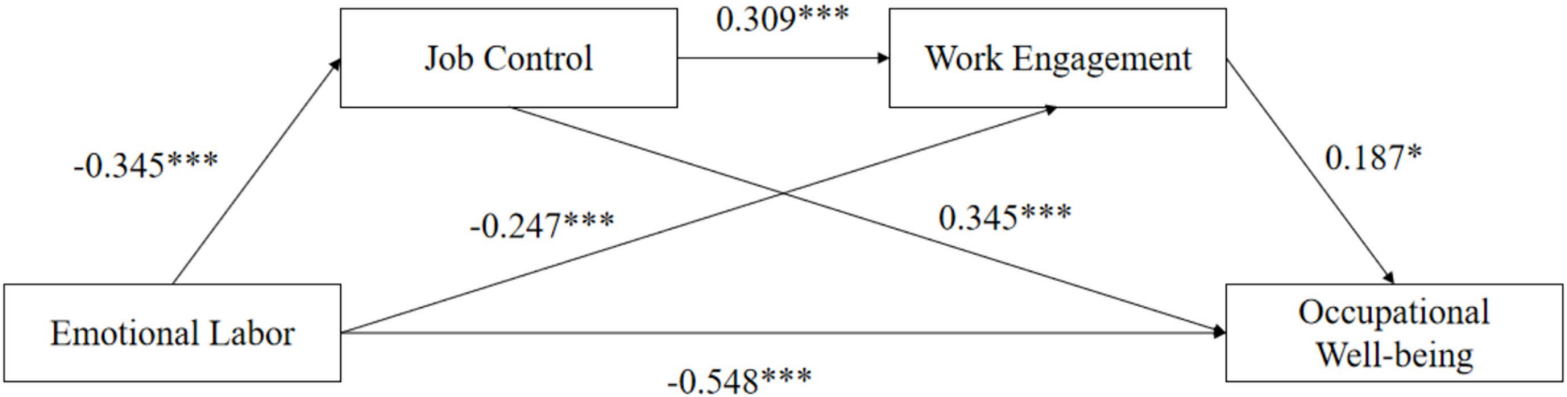
Serial mediation model with standardized path coefficients. *p < 0.05, **p < 0.01, ***p < 0.001.

A serial mediation analysis was conducted using bootstrap procedures with 5,000 resamples. Emotional labor was specified as the independent variable, occupational well-being as the dependent variable, and job control and work engagement as sequential mediators. The results revealed that all indirect pathways were statistically significant (Table 4; Figure 2). Overall, these findings indicate that emotional labor is associated with occupational well-being both directly and indirectly through independent and sequential correlational pathways involving job control and work engagement.

**Table 4:** Results of the serial mediation analysis.

Effect	Effect estimate	Bootstrap SE	95% Boot CI lower	95% Boot CI upper	*P*
Indirect effect 1	−0.119	0.032	−0.182	−0.056	0.001
Indirect effect 2	−0.046	0.023	−0.091	−0.001	0.045
Indirect effect 3	−0.107	0.029	−0.163	−0.051	0.001
Total indirect effect	−0.272	0.055	−0.380	−0.164	0.001
Direct effect	−0.548	0.099	−0.742	−0.354	0.001
Total effect	−0.820	0.094	−1.005	−0.635	0.001

### 3.6 Moderated serial mediation analysis

The moderating role of organizational support in the proposed model was further examined. As shown in Table 5, emotional labor had a significant negative effect on job control (β = −0.347, *p* < 0.001). The interaction term between emotional labor and organizational support was statistically significant (β = 0.018, *p* < 0.001), indicating that organizational support significantly moderates the relationship between emotional labor and job control. Specifically, higher levels of organizational support attenuated the negative effect of emotional labor on job control.

**Table 5:** Results of the moderated serial mediation analysis.

	(1)	(2)	(3)	(4)
Variables	Occupational well-being	Job control	Work engagement	Occupational well-being
Emotional labor	−0.733^***^	−0.347^***^	−0.247^**^	−0.548^***^
	(−6.539)	(−6.668)	(−3.067)	(−4.889)
Organizational support		−0.226^***^		
		(−4.042)		
Emotional labor* Organizational support		0.018^***^		
		(16.830)		
Job control			0.309^***^	0.345^***^
			(5.530)	(4.303)
Work engagement				0.187^*^
				(2.573)
Constant	119.303^***^	57.820^***^	27.414^***^	82.426^***^
	(21.290)	(19.128)	(4.656)	(9.874)
*N*	364	364	364	364
*R*^2^	0.106	0.529	0.124	0.183

*p < 0.05, **p < 0.01, ***p < 0.001.

**Figure 3:**
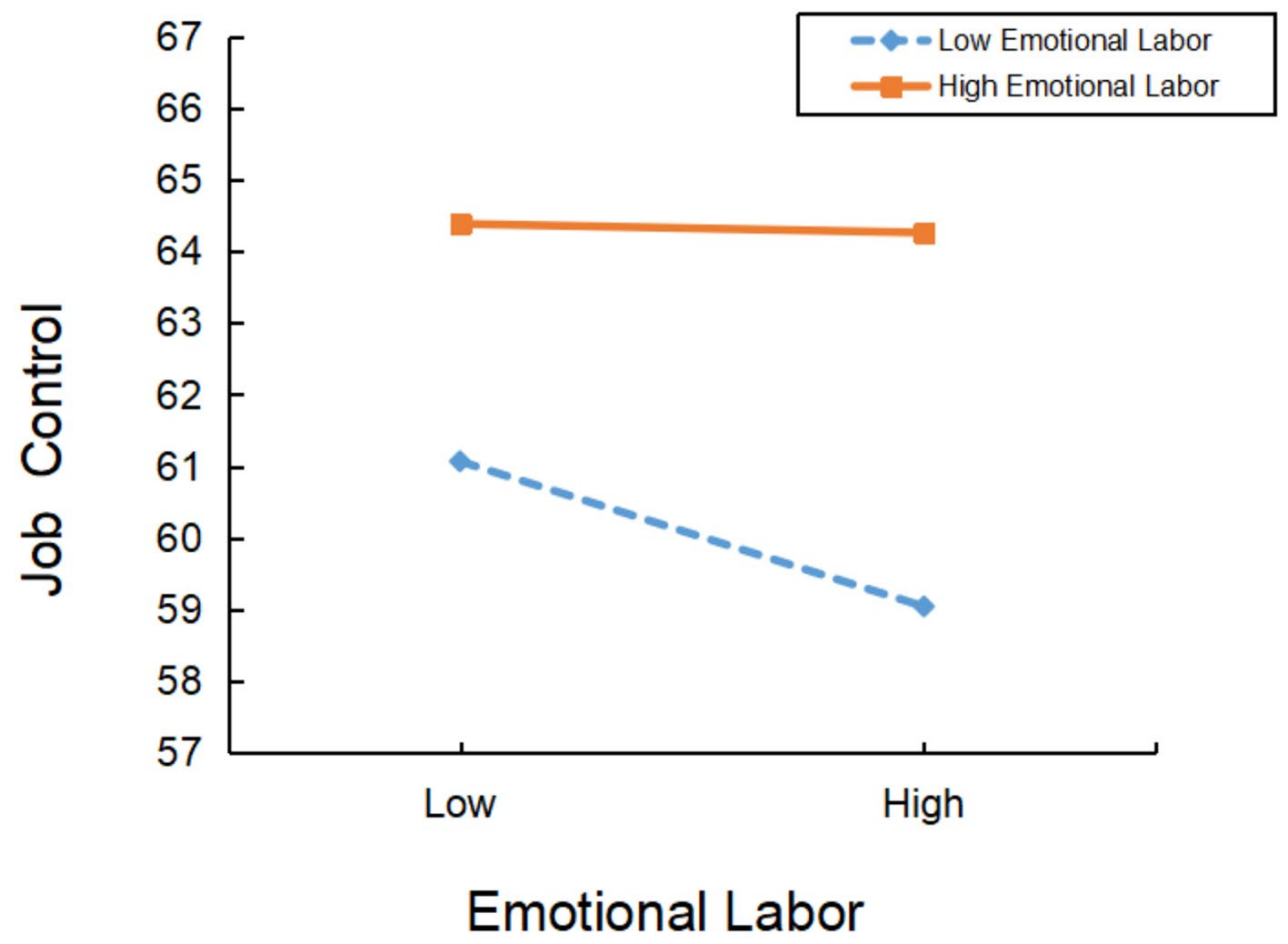
Moderating role of organizational support.

To further interpret the interaction effect, a simple slope analysis was conducted. The results showed that at low levels of organizational support, emotional labor had a significant negative effect on job control (β = −0.757, *t* = −4.220, *p* = 0.001). In contrast, at high levels of organizational support, this negative effect was weakened and became non-significant (β = −0.293, *t* = −1.810, *p* = 0.072). These findings suggest that organizational support buffers the adverse impact of emotional labor on job control (Figure 3).

Taken together with the mediation results, these findings provide preliminary support for the proposed moderated mediation relationship, in which organizational support weakens the initial stage of the mediation process between emotional labor and job control, thereby influencing the overall indirect effect on occupational well-being.

## 4 Discussion

### 4.1 Main findings

This study examined the relationship between emotional labor and occupational well-being among endoscopy healthcare workers and further explored the underlying mechanisms using a moderated serial mediation model. The results showed that emotional labor was significantly negatively associated with occupational well-being. In addition, job control and work engagement played both independent and sequential mediating roles in this relationship, while organizational support moderated the association between emotional labor and job control.

These findings provide empirical support for the proposed theoretical model and contribute to a more comprehensive understanding of how emotional labor relates to occupational well-being in healthcare settings.

### 4.2 The association between emotional labor and occupational well-being

This study found that emotional labor is significantly negatively associated with occupational well-being among endoscopy healthcare workers. This finding is consistent with previous studies (34) and can be interpreted within the Job Demands–Resources (JD-R) model and Conservation of Resources (COR) theory (35). This result supports Hypothesis 1 (H1).

According to the JD-R model, emotional labor can be regarded as a continuous job demand that requires healthcare workers to regulate their emotional expressions during patient interactions. COR theory further explains why such emotional demands may be associated with reduced occupational well-being: individuals strive to preserve valuable psychological resources, but repeated emotional regulation may gradually consume these resources and increase the risk of resource loss (14, 36). In the context of endoscopy centers, healthcare professionals are required not only to perform high-precision technical procedures but also to continuously regulate their emotions in response to patients and their families, resulting in the accumulation of both technical and emotional demands (37). When these demands exceed available resources, individuals may experience reduced positive work experiences, lower job satisfaction, and a weakened sense of professional achievement (38).

Moreover, emotional labor is characterized by its pervasive nature, extending throughout the entire process of doctor–patient interactions, and is often difficult for organizations to directly recognize or compensate (39). When individuals are exposed to the dual burden of task execution and emotional regulation over a prolonged period, they are more likely to experience a sense of work imbalance and develop less positive evaluations of their profession (40). Combined with the high-paced and high-risk nature of endoscopy center work, the negative effects of emotional labor may be further amplified. Therefore, the findings of this study reflect the cumulative adverse impact of emotional labor on occupational well-being in high-demand work environments.

### 4.3 The serial mediation mechanism of job control and work engagement

The results of this study indicate that job control and work engagement play significant mediating roles in the relationship between emotional labor and occupational well-being, and together form a serial mediation pathway, thereby jointly supporting Hypotheses 2, 3, and 4 (H2–H4). This finding further elucidates the underlying mechanism through which emotional labor relates to occupational well-being from the integrated perspective of the JD-R model and COR theory (41).

The JD-R model provides a useful framework for positioning emotional labor as a job demand and job control as an important job resource. However, COR theory offers a more specific explanation of the sequential process proposed in this study. From the perspective of COR theory, continuous emotional regulation may first consume individuals’ psychological and cognitive resources, thereby weakening their perceived control over work tasks and work processes. Reduced job control may further limit individuals’ ability to mobilize energy and motivation at work, resulting in lower work engagement. This resource depletion process helps explain why job control and work engagement function as sequential mediators between emotional labor and occupational well-being. Previous studies have similarly shown that high emotional demands and emotional strain may weaken healthcare professionals’ positive work states and exacerbate psychological resource depletion (42). In contrast, higher levels of autonomy and job control are closely associated with more favorable work outcomes (43).

Furthermore, reduced job control may negatively affect work engagement. According to the motivational pathway of the JD-R model, job resources are essential for fostering work engagement. When individuals perceive higher levels of control, they are more likely to experience vigor, dedication, and absorption; conversely, insufficient job resources may suppress their level of engagement (44). From the perspective of COR theory, job control can also be viewed as an important psychological resource. When this resource is weakened under sustained emotional labor demands, individuals may become more cautious in allocating their remaining energy, thereby reducing their work engagement. At the same time, work engagement is an important psychological mechanism underlying differences in occupational well-being. Higher levels of work engagement are generally associated with better quality of working life and greater occupational well-being (45). Studies among nursing populations have similarly shown that work engagement enhances psychological well-being and mediates the relationship between resource factors and well-being outcomes (46). The serial mediation pathway identified in this study suggests that emotional labor does not influence occupational well-being through a single pathway but rather exerts its negative effects through a progressive process of resource depletion. Specifically, emotional labor first diminishes individuals’ sense of job control, which in turn affects their level of work engagement, ultimately leading to reduced occupational well-being. This finding highlights the critical mediating role of psychological resources in linking job demands to occupational outcomes.

### 4.4 The moderating role of organizational support

The results of this study indicate that organizational support significantly moderates the relationship between emotional labor and job control, such that organizational support buffers the negative impact of emotional labor on job control. This finding supports Hypothesis 5 (H5).

Within the framework of the JD-R model, organizational support represents an important job resource that may help employees cope with emotional demands and maintain a sense of control over their work (42). COR theory further suggests that external resources provided by the organization may compensate for resource loss and protect individuals from further depletion. Therefore, when healthcare professionals perceive higher levels of organizational support, they may be more likely to obtain emotional support, institutional protection, and resource supplementation, which helps them maintain job control when facing emotional labor demands (47, 48). In contrast, when organizational support is insufficient, emotional labor may more easily translate into perceived resource loss and reduced job control.

From the perspective of social exchange theory, when individuals perceive that the organization values and supports them, they are more likely to develop positive psychological expectations and work attitudes, which enhances their ability to adapt to the work environment (49). In this process, organizational support not only directly provides resources but also indirectly strengthens individuals’ ability to maintain job control by enhancing their sense of psychological safety and belonging (50).

Therefore, the findings of this study suggest that organizational support plays a crucial buffering role in the relationship between emotional labor and job control. In other words, under conditions of high organizational support, the negative effect of emotional labor on job control is significantly weakened, whereas under conditions of low organizational support, this negative effect becomes more pronounced. This finding further highlights the critical role of organizational resources in regulating the relationship between job demands and individuals’ psychological responses.

### 4.5 Practical implications

From a practical perspective, these findings suggest that improving occupational well-being among endoscopy healthcare workers requires systematic optimization that balances job demands and job resources.

First, efforts should be made to reduce unnecessary emotional labor demands by optimizing work processes and staffing allocation, thereby minimizing the excessive consumption of individuals’ psychological resources caused by high job demands. Second, greater attention should be paid to the development of key job resources. In particular, enhancing medical staff’s participation in work arrangements and decision-making processes can improve their level of job control, while well-designed incentive and support mechanisms can promote higher levels of work engagement, thereby sustaining positive work states.

At the organizational level, it is also essential to strengthen the development of a supportive work environment by providing emotional support and institutional safeguards, which can buffer the negative impact of emotional labor on individuals’ perceived resources. In contexts where high job demands are unavoidable, only by simultaneously reducing excessive demands and enhancing multi-level job resources can the adverse effects of emotional labor on occupational well-being be effectively mitigated.

### 4.6 Limitations

This study has several limitations. First, a cross-sectional design was adopted, which allows for the identification of associations among variables but does not permit causal inferences.

Second, the data in this study were primarily collected through self-reported questionnaires at a single time point. Although common method bias was preliminarily assessed using Harman’s single-factor test, this method alone cannot completely eliminate the possibility of common method variance, and potential bias may still exist.

In addition, this study used a convenience sampling method, and the sample was mainly drawn from hospitals in selected regions, which may limit the generalizability of the findings.

Finally, this study only examined the mediating roles of job control and work engagement, as well as the moderating role of organizational support, without considering other potential influencing factors. In addition, although this study examined the moderating role of organizational support in the relationship between emotional labor and job control, conditional indirect effects and the index of moderated mediation were not further estimated. Therefore, the moderated indirect pathway should be interpreted with caution.

### 4.7 Future research directions

Based on the findings of this study, future research can be further expanded in several directions. First, in terms of research design, longitudinal or experimental approaches may be employed to more thoroughly examine the causal relationship between emotional labor and occupational well-being, as well as their dynamic changes over time.

Second, regarding data sources and methodological rigor, future studies may incorporate multi-source data, such as supervisor evaluations and objective work-related indicators, and adopt longitudinal designs to reduce the potential bias associated with single-source self-reported data. Although confirmatory factor analysis was conducted to examine construct validity, future studies may further strengthen methodological rigor by incorporating longitudinal designs and multi-source data.

Third, in terms of variable expansion, future research could extend the current model by incorporating additional types of job resources, organizational factors, or individual characteristics. In particular, future studies may further examine the potentially different roles of emotional labor subdimensions, including surface acting, deep acting, and natural expression, to provide a more comprehensive understanding of the mechanisms linking emotional labor and occupational well-being. In addition, future studies may further estimate conditional indirect effects and the index of moderated mediation to more comprehensively examine the moderated indirect pathways underlying emotional labor and occupational well-being.

Furthermore, as this study focused on endoscopy healthcare workers, future research could extend this work to other clinical settings to further examine the generalizability and contextual robustness of the findings. This would also enrich the application of the integrated JD-R and COR framework in healthcare settings and allow for a more in-depth examination of how job demands, job resources, and individual resource conservation processes jointly shape occupational well-being.

## Data Availability

The raw data supporting the conclusions of this article will be made available by the authors, without undue reservation.
